# Context is Queen: The Good, the Bad and the Rugby: Understanding Engagement With Injury Surveillance in Women's Rugby Union

**DOI:** 10.1002/ejsc.70230

**Published:** 2026-09-16

**Authors:** Molly F. McCarthy‐Ryan, Elin James, Stephen D. Mellalieu, Gemma M. Robinson, Simon Roberts, Debbie Palmer, Isabel S. Moore

**Affiliations:** ^1^ Cardiff School of Sport and Health Sciences Cardiff Metropolitan University Cardiff UK; ^2^ UK Collaborating Centre on Injury and Illness Prevention in Sport Universities of Edinburgh and Bath Edinburgh and Bath UK; ^3^ Centre for Health, and Injury & Illness Prevention in Sport Department of Health University of Bath Bath UK; ^4^ Edinburgh Sports Medicine Research Centre Institute for Sport PE and Health Sciences University of Edinburgh Edinburgh UK

**Keywords:** community, injury surveillance, intrinsic motivation, rugby union, self‐determination theory

## Abstract

To address the gender imbalance in rugby union research by exploring barriers and facilitators to injury surveillance participation among female community players, thereby providing insight into how psychological needs shape engagement. This study examines how knowledge of injury surveillance and practical barriers influence engagement with injury surveillance systems in women's community rugby. Cross‐sectional. An online survey was completed by 242 women's community rugby union players (age: 29 ± 9 years; experience: 7.1 ± 5.2 years) from England, Ireland, Scotland and Wales. The questionnaire covered demographics, rugby environment, injury surveillance knowledge, and motivation to play rugby (adapted SMS‐II). Barriers and facilitators were rated on a 7‐point Likert scale. Data were analysed using descriptive statistics, Kruskal–Wallis tests, regression models and chi‐square tests (SPSS v29; *p* < 0.05). Coach presence was high (> 94%), but medical support was limited (26% training; 49% matches). Women's community rugby was perceived as the lowest priority for NGBs. Earlier rugby initiation predicted greater experience (*p* = 0.002) and higher likelihood of injury surveillance collection (OR = 1.23 per year; *p* = 0.012). Perceived lack of expertise reduced engagement (*p* = 0.024). Intrinsic motivation negatively predicted playing experience (*p* = 0.048), whereas agreement with ‘I just want to play rugby’ was associated with lower surveillance participation (*p* = 0.013). Engagement with injury surveillance is shaped by motivation, experience and structural support. Athlete‐centred strategies, such as inclusive policy, tailored education and female‐specific data systems, are essential to improve player welfare and advance evidence‐based practice in women's rugby union.

## Highlights

- Players who started rugby before age 18 were more likely to engage in injury surveillance, highlighting the importance of early participation for fostering proactive health behaviours.
- Limited medical support at community level often forces coaches to manage injuries, underscoring the need for dedicated medical personnel to protect player welfare.
- Engagement with injury surveillance is strongly linked to perceived competence and autonomy, suggesting that education and tools should be simple, inclusive, and athlete centred.

## 1 Introduction

Despite the increasing participation of women in rugby union, the sport science and sports medicine literature continue to be disproportionately focused on male athletes. This gender imbalance has led to a limited understanding of the distinct physiological, psychological, and sociocultural factors influencing female athletes participation in rugby union, particularly at the community level (J. T. Costello et al. 2014; Heyward et al. 2022; Moore et al. 2024). Injury surveillance represents a component of evidence‐based practice within sport, providing the data necessary to inform injury preventions strategies and policy development. Injury surveillance research is lacking in female rugby cohorts, with minimal data available in the community side of the game (Walshe et al. 2024). This gap presents a barrier to the development of effective and contextually relevant injury prevention strategies. Implementing injury surveillance in community rugby settings is complex and often hindered by logistical, financial, and cultural constraints. These challenges are compounded by broader issues of representation and institutional support. National governing bodies (NGBs) play a critical role in shaping the visibility and legitimacy of women's sport. When female athletes are underrepresented in research and policy, it can negatively impact their social identity, sense of belonging, and engagement with health and safety initiatives (Allen et al. 2021; Stevens et al. 2021; Mkumbuzi 2024), such as community injury surveillance systems.

Sport injury prevention frameworks such as Translating Research into Injury Prevention Practice framework (TRIPP; Finch 2006) emphasise the importance of injury surveillance systems to help establish the injury problem and identify injury mechanisms before implementing interventions. Yet, foundational data on injury rates in female athletes is often missing, particularly in community settings. Advocacy for female‐specific research has historically been weak, and funding remains disproportionately allocated to male‐focused studies (Emmonds et al. 2019). Several countries implement women's rugby community injury surveillance, but typically the injury surveillance systems lack adaptations to accurately capture data relevant to female rugby players (e.g., non‐time loss data (Moore et al. 2024)) that goes beyond an injury and includes a focus on female health problems (Fuller et al. 2007; Yeomans et al. 2021; Starling et al. 2023; Moore et al. 2024). Beyond structural barriers, psychological and motivational factors may also influence engagement with injury surveillance, which can occur at multiple levels. Typically, most systems require a club designate to collate and report team data to the research team, making their engagement critical. Player engagement is also essential, whether to provide consent, report injuries to the club designate, or self‐report if the system requires it (D. Costello et al. 2024). Self‐Determination Theory (SDT) offers valuable insights into why female athletes may be reluctant to participate in surveillance programs. SDT highlights three core psychological needs: autonomy, competence, and relatedness, which are essential for motivation and well‐being (Ryan and Deci 2000). In environments where athletes feel excluded from decision‐making or overwhelmed by technical systems, these needs may be unmet, leading to reduced engagement or even amotivation.

Emerging evidence suggests that an individuals' belief in their capability to collect injury surveillance, often referred to as ‘perceived competence’, may influence motivation to engage with injury surveillance more strongly than actual technical accuracy (Vella et al. 2025).This is particularly relevant in community‐level women's rugby union, where formal education around injury surveillance may be limited, and players rely more on intuitive understanding and peer support (Walton et al. 2025). Motivation may also vary depending on how the injury surveillance is framed and understood, with recreational environments fostering intrinsic motivation through enjoyment and social connection (Ryan and Deci 2020). This study is the first to systematically assess the perceived barriers and facilitators to injury surveillance participation among female rugby union athletes. By applying SDT, it explores how different types of motivation to participate in sport, ranging from intrinsic to external regulation, interact with players' understanding of injury surveillance, and how players' understanding, perceived barriers, and contextual relevance shape engagement with surveillance. Specifically, to address the overarching aim of understanding injury surveillance engagement, this study seeks to: (1) examine how knowledge of injury surveillance influences the proportion of female community teams that collect rugby injury surveillance data, and (2) identify the perceived barriers and facilitators to implementing injury surveillance in women's rugby union.

## 2 Methods

Ethical approval was obtained under the Cardiff Metropolitan University Ethics Framework, alongside letters of support from the four nations' rugby governing bodies.

### 2.1 Participants

A total of 242 women's community rugby union players (mean age: 29 ± 9 years; playing experience: 7.12 ± 5.17 years) completed an online survey after providing voluntary, informed consent. Eligible participants were female rugby union players aged 18 years or older, residing in England, Ireland, Scotland, or Wales, and actively playing rugby union at any level within the UK or Ireland. All four nations actively undertake women's rugby community injury surveillance. The term ‘female’ refers to sex assigned at birth, in accordance with World Rugby's policy. The term ‘women’ is used to describe biologically female rugby players participating in the women's game. All data were anonymised and stored on a general data protection regulation compliant online system. This international research project was supported by the English Rugby Football Union, Irish Rugby Football Union, Scottish Rugby Union and Welsh Rugby Union.

### 2.2 Equity, Diversity, and Inclusion Statement

The research team comprised five women (including first and senior authors) and two men, consisting of junior (2), mid‐career (1) and senior researchers (4). Although from different disciplines, all authors were from the United Kingdom. The study focuses on sex and discusses gender in its context but does not address socioeconomic status or race/ethnicity.

### 2.3 Survey

This observational, cross‐sectional study was designed in accordance with the CHERRIES guidelines (Eysenbach 2004). An electronic questionnaire was developed through roundtable discussions involving experts in sport performance analysis (GR), sports psychology (SM), epidemiology, and human movement science (SR, DP, MMR, EJ, ISM). Items were drafted to address the research aims by MMR, GR, SM and ISM. Item development was informed by reviewing the literature, specialism expertise and lived experience conducting community injury surveillance and discussions with community players. All authors then reviewed the items as well as representatives (*n* = 5) from the community rugby union game, who also piloted the survey to ensure relevance and clarity. Minor amendments to the phrasing of questions were completed, but no items were added or removed. Survey functionality was tested on mobile phones and computers using Qualtrics (www.qualtrics.com; version June 2024). The survey was built and hosted on Qualtrics, using a bespoke survey URL which was generated and distributed via social media platforms, researcher networks (e.g., Welsh Rugby Players' Association), and word of mouth. The survey remained open for one season, from 1st August 2024 to 1st August 2025. The questionnaire was structured into four key sections: (1) demographics, (2) rugby environment, (3) injury surveillance knowledge, and (4) participation motivation. The demographics section included 11 closed‐ended items capturing variables such as playing level and years of experience. The rugby environment section comprised 26 closed‐ended questions addressing weekly training frequency, coaching support, and access to facilities, including club infrastructure and female‐specific hygiene provisions. Injury surveillance knowledge was assessed through 10 closed‐ended items. Participants were asked to identify the correct definition of sports injury surveillance, indicate whether their rugby team engages in injury data collection, and to rate perceived barriers and facilitators to implementing injury surveillance practices. Barriers and facilitators were presented for rating from 1 (strongly disagree) to 7 (strongly agree). The statements assessed were as follows: It is difficult to integrate injury surveillance because it takes time; It is useless to carry out injury surveillance as injuries can't be prevented (e.g., an uncontrolled external event); I am not sure that injury surveillance would be useful for me; I just want to play rugby; There is no‐one with enough expertise to manage the data collection required for injury surveillance at my team; Workshops on injury surveillance; Videos on injury surveillance; Appropriate staffing in the team; Increase budget to support engagement in injury surveillance; Team specific feedback on my team's injuries; Knowing data is being used to inform policies. Participation motivation was evaluated using a version of the Revised Sport Motivation Scale (SMS‐II; Pelletier et al. 2013), adapted from general sport to be rugby‐specific, designed to assess players' motivational profiles in relation to rugby union participation.

### 2.4 Data Extraction and Statistical Analysis

Only fully completed surveys from eligible participants were analysed to ensure the sample size was consistent across all statistical tests performed. Based on the number of participants who consented (*n* = 258), there was a completeness rate of 94%. Given the possibility of players completing the survey multiple times, duplicates were checked by team, age, nationality, playing position and level. No duplicates were recorded. Descriptive statistics were computed to summarise participant demographics. Differences in playing experience across categories of rugby initiation age were assessed using the Kruskal–Wallis test. Where significant effects were observed, post hoc pairwise comparisons were conducted using Mann–Whitney U tests with Bonferroni adjustment.

Linear regression analysis was employed to examine associations between playing experience and motivation‐related variables, with all variables entered simultaneously. Prior to analysis, multicollinearity was satisfactory, with all tolerance values > 0.1 and VIF < 10. Inspection of the normal P‐P plot indicated that residuals were approximately normally distributed. Only significant predictors are reported. Binary logistic regression was used to identify predictors of injury surveillance collection. Again, all variables were entered simultaneously. Prior to analysis, assumptions were assessed and satisfied: no multicollinearity (tolerance > 0.1, VIF < 10), linearity of the logit confirmed (Box‐Tidwell, *p* = 0.876), and adequate model fit (likelihood ratio *p* = 0.002, Pearson goodness‐of‐fit *p* = 0.998). Twelve influential cases were identified via Cook's distance and are acknowledged as a limitation. Associations between perceived barriers and facilitators to injury surveillance collection were evaluated using chi‐square tests. Chi‐square tests examining perceived barriers and facilitators were considered exploratory; correction for multiple comparisons was therefore not applied in order to minimise the risk of Type II error. Full regression analysis details are available in Supporting information S1. All statistical analyses were performed using SPSS Statistics (Version 29.0; IBM Corp., Armonk, NY, USA), with significance set at *p* < 0.05.

## 3 Results

### 3.1 Demographics Characteristics

Table 1 presents the demographic characteristics of the 242 participants. Coach *presence* was consistently reported with only 1% (*n* = 3) and 6% (*n* = 15) of players indicating that a coach was present only occasionally during training and matches, respectively; one participant reported having no coach present at matches. In contrast, access to medical personnel was notably lower, with 26% and 49% of players reporting their presence at training and matches, respectively, and 25% reporting no medical personnel being present.

**TABLE 1:** Demographic data for participants, displayed as mean ± standard deviation, or *n* (%).

Variable	Level	Mean ± SD, or *n* (%)
Age		29 ± 9 years
Playing experience		7.1 ± 5.2 years
Player level	Amateur/community	159 (66%)
	Club/university	83 (34%)
Started playing	Club (< 18)	50 (21%)
	Club (> 18)	116 (48%)
	School	34 (14%)
	University	42 (17%)
Training sessions per week	1–2	170 (70%)
	3–4	51 (21%)
	4+	21 (9%)

**FIGURE 1:**
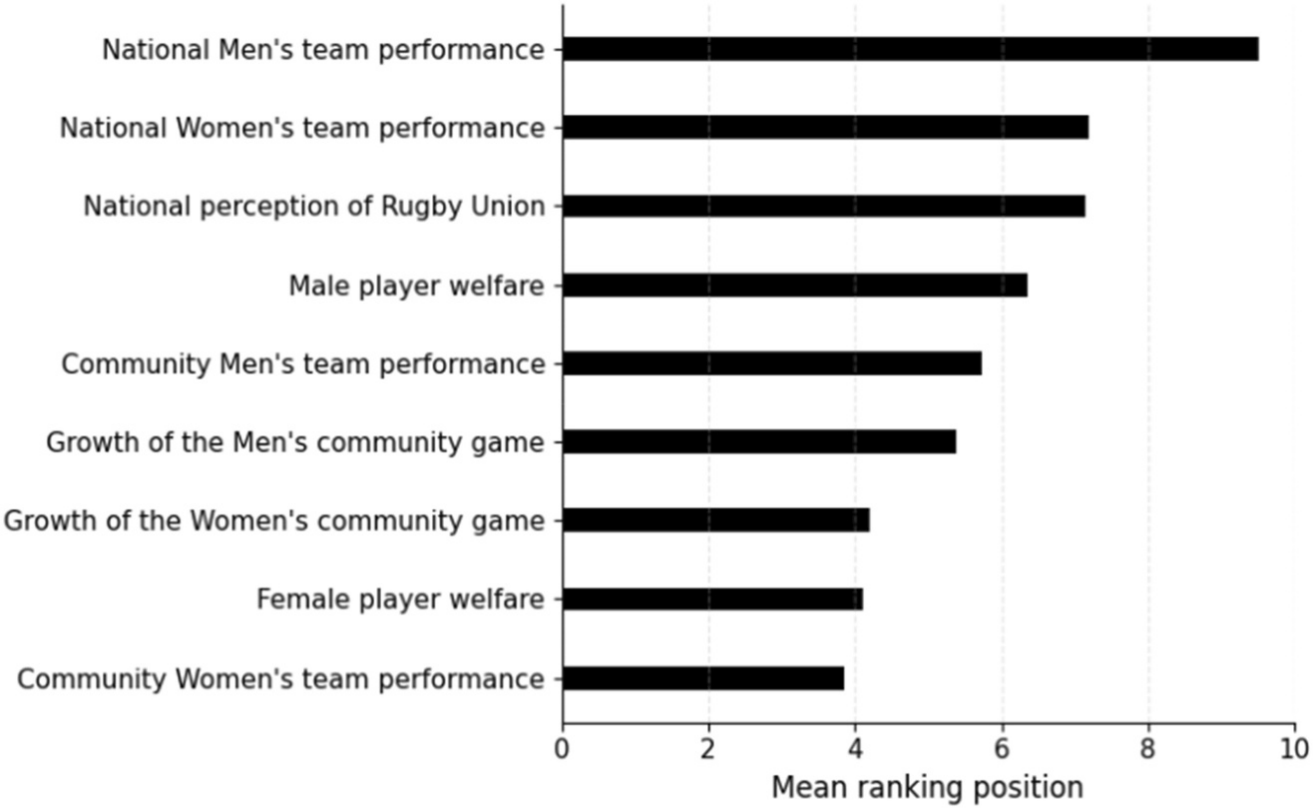
Ranked areas of rugby in order of perceived priority by the NGB.

When asked to rank perceived priorities of their NGB, participants identified the men's national team as most prioritised, followed by the women's national team, the men's community game, and lastly the women's community game, including aspects of player welfare, development, and performance (Figure 1).

### 3.2 Playing Experience and Expertise

**FIGURE 2:**
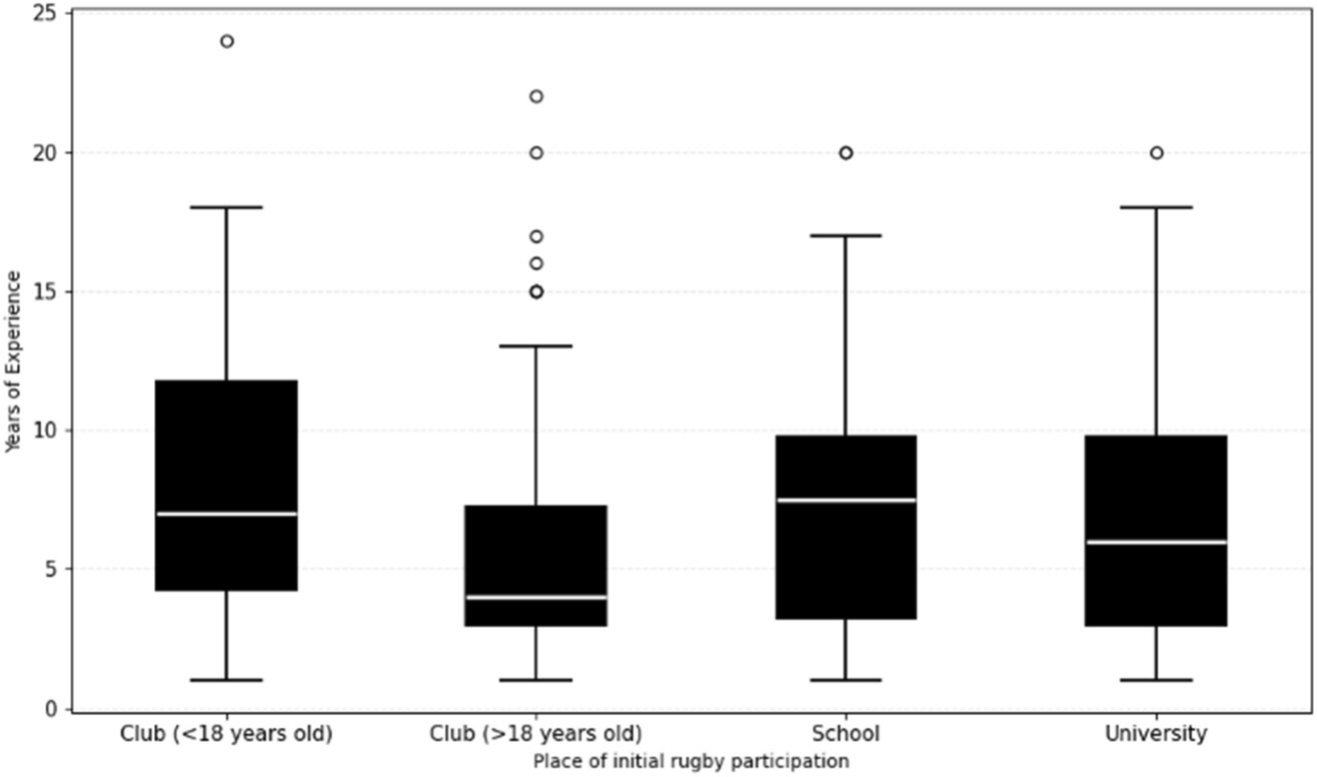
Playing experience across settings of initial rugby participation. The median is highlighted by a white line. Outliers are plotted as individual points.

There was a difference in playing experience across categories of rugby initiation age (*χ* ^2^(3, *N* = 242) = 14.55, *p* = 0.002; Figure 2). Post hoc pairwise comparisons indicated that players who began playing at club level before the age of 18 had greater playing experience than those who started at club level after 18 (*U* = 1997, *p* = 0.001). Furthermore, players who initiated rugby at club level after 18 had less experience than those who began at school (*U* = 1392, *p* = 0.009), but more than those who started at university (*U* = 1911, *p* = 0.038).

Playing experience predicted injury surveillance collection by their team (*B* = 0.205, *p* = 0.012), with each additional year of experience associated with a 23% increase in the odds of the team collecting injury surveillance (Exp(*B*) = 1.227). The full regression model explained 54.3% of the variance in injury surveillance collection (Nagelkerke *R* ^2^ = 0.543). Participants who began at a club before age 18 were more likely to collect injury surveillance (*B* = −3.647, *p* = 0.044, Exp(*B*) = 0.026) than those who started at university.

**FIGURE 3:**
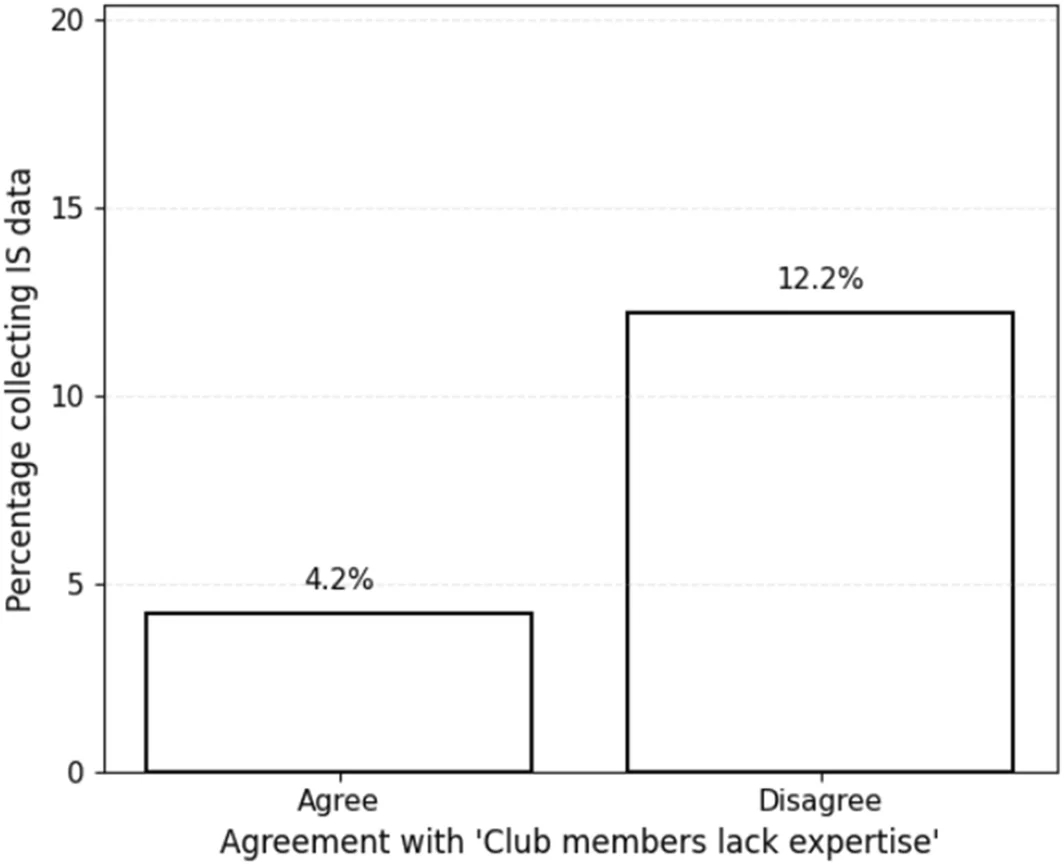
Proportion of respondents who reported lack of personnel in the team for collecting injury surveillance (IS) data.

There was an association between agreement that club members do not have enough expertise for data collection and injury surveillance collection (*χ* ^2^(1, *n* = 242) = 5.10, *p* = 0.024; Figure 3). Those who disagreed were more likely to collect injury surveillance (12.2%) than those who agreed (4.2%).

### 3.3 Players' Motivation to Play Rugby Union

Analysis of self‐regulation in rugby union players revealed that intrinsic motivation was the only predictor of years spent playing (playing experience), with higher intrinsic motivation associated with lower experience (*B* = −2.383, 95% CI [−4.716, −0.050], *p* = 0.048). However, the overall model was non‐significant (F(7, 234) = 1.602, *p* = 0.136, *R* ^2^ = 0.046), so this should be interpreted with caution. No significant associations were found between playing experience and other motivational constructs, including integrated, identified, introjected, external, and non‐regulation forms of motivation.

**FIGURE 4:**
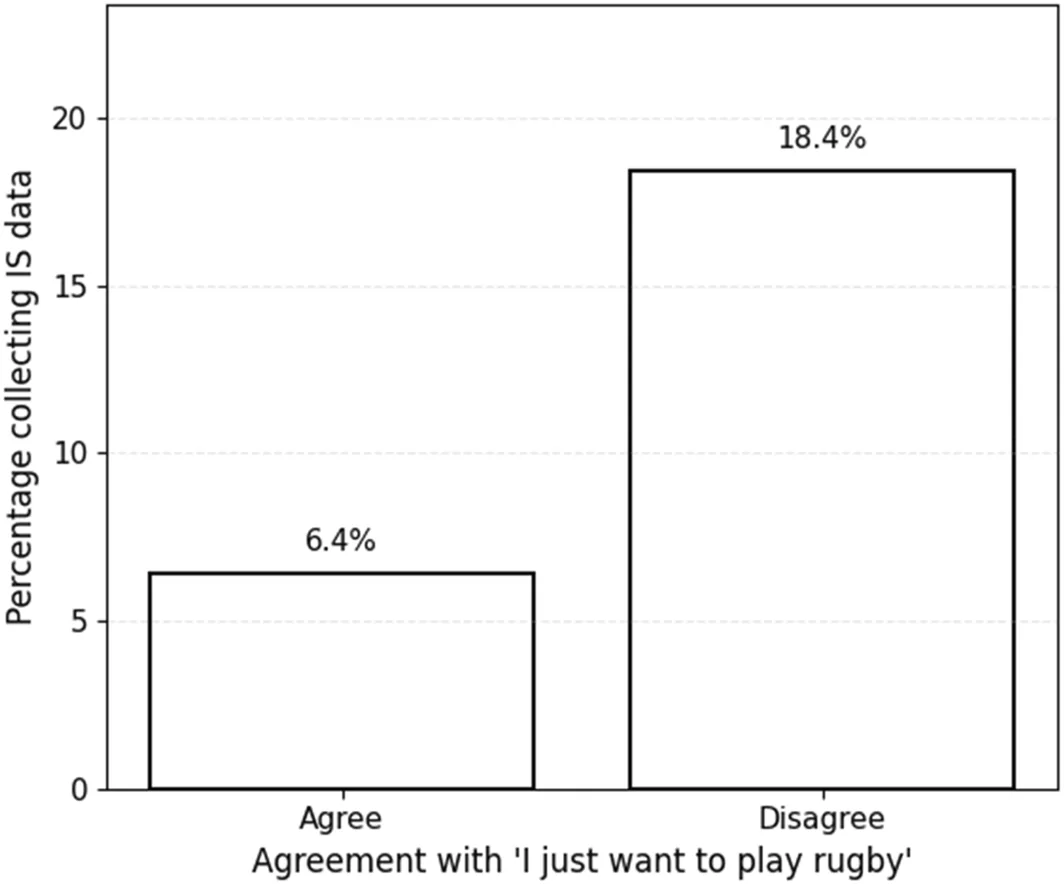
Proportion of respondents identifying a desire to ‘just play rugby’ to collecting injury surveillance (IS) data.

There was an association between agreement with *‘I just want to play rugby’* and whether participants collect injury surveillance data (*χ* ^2^(1, *n* = 242) = 6.13, *p* = 0.013; Figure 4), with participants who disagreed more likely to be part of a team which collects injury surveillance data (18.4%) compared to those who agreed (6.4%).

## 4 Discussion

The study sought to: (1) examine how knowledge of injury surveillance influences the proportion of female community teams that collect rugby injury surveillance data, and (2) identify the perceived barriers and facilitators to implementing injury surveillance in women's rugby union. The findings revealed that players who began playing rugby before the age of 18 tended to have greater playing experience and were more likely to engage in injury surveillance practices. Conversely, higher intrinsic motivation was associated with less playing experience. Importantly, players who identified sports injury prevention as important were significantly more likely to engage with injury surveillance data collection. These insights suggest that early engagement in the sport and a strong awareness of injury prevention may contribute to more consistent adoption of evidence‐based approaches, such as injury surveillance, at the community level.

### 4.1 Playing Experience and Engagement With Injury Surveillance

Players who began playing rugby before the age of 18 were more likely to have greater playing experience and to engage in injury surveillance activities, suggesting that early sport participation may be linked to increased sports‐related injury awareness and proactive health behaviours. This pattern supports the role of autonomous motivation in promoting proactive health management (Chiu et al. 2023). Interestingly, higher intrinsic motivation was associated with less playing experience. This indicates that newer players may be driven more by enjoyment and personal growth than by performance or safety concerns. This aligns with SDT, which posits that intrinsic motivation is rooted in personal satisfaction rather than external outcomes (Ryan and Deci 2000, 2020). Players who began playing rugby before the age of 18 had accumulated more years of playing experience, suggesting that early engagement is linked to sustained participation. This trend also reflects broader patterns identified in our study and in Williams et al. (2022). These findings highlight that many women enter rugby later in life, often at university or club level, resulting in fewer years of experience, despite their current age. Furthermore, our results show, while coaches were consistently present at both training sessions and matches, medical personnel were frequently absent. As a result, community rugby coaches frequently took on additional roles, including injury management, despite lacking formal medical training (Roberts et al. 2013). This dual responsibility may be likely to place pressure on coaches and may compromise player welfare, especially in high‐risk environments with limited medical oversight. Collectively, these findings suggest that early sport participation is associated with increased injury awareness and proactive health behaviours. Ensuring community rugby is adequately resourced in terms of medical personnel should be prioritised for player welfare purposes. This may also be associated with greater women's rugby community engagement with injury surveillance.

Engagement with injury surveillance in community‐level women's rugby union may be influenced by athletes' perceived capability and understanding of injury surveillance processes, particularly in informal environments where structured education is limited (Walshe et al. 2024). This reflects Deci's (1971) undermining effect, where external controls, such as rigid systems or imposed expectations, can reduce intrinsic motivation, especially when autonomy is compromised. The undermining effect of intrinsic motivation also suggests that external rewards for initially intrinsically motivated behaviour can reduce their intrinsic motivation (Deci 1971). Newer players often participate in *sport* for enjoyment and social connection, satisfying both autonomy and relatedness (Pope and Wilson 2012). As experience increases, motivations may shift toward more external drivers, such as performance goals, leadership responsibilities, or team obligations. This transition may explain why more experienced players are more likely to engage with injury surveillance practices. Their motivation to play may become increasingly identified or integrated with team and performance values (Kim et al. 2023; Barbosa Cano and Gomez‐Baya 2025). In environments where athletes feel excluded from decision‐making processes or overwhelmed by complex technical systems, these needs may be thwarted. This may lead to disengagement or amotivation (Chiu et al. 2023). Therefore, creating conditions that support athletes' sense of capability, autonomy, and connection to the broader purpose of rugby may play a role in enhancing meaningful engagement with IS and other welfare‐related practices.

### 4.2 Motivators for Participation and Engagement With Injury Surveillance

Motivational dynamics were further reflected in players' responses to specific survey items. Those who disagreed with the statement *‘I just want to play rugby’* were more likely to engage in injury surveillance data collection. This suggests that players who perceive their role as extending beyond personal enjoyment tend to be more engaged with practices that support the sport's development. This aligns with findings from Kim et al. (2023), who identified empowerment, challenge, and a desire to give back to the sport as key intrinsic motivators among female rugby players. However, many sports injury surveillance systems remain designed around male athletes and are not adapted sufficiently to capture data relevant to female rugby players (Moore et al. 2024). This design bias is associated with slower progress in sex‐specific policy development and limits institutional support from NGBs. Advocacy for female‐specific research has historically been weak, with funding disproportionately allocated to male‐focused studies (Emmonds et al. 2019). These structural and psychological barriers collectively undermine female athletes' social identity and psychological well‐being (Pascoe et al. 2022). When athletes feel undervalued or unsupported, lower engagement with health and safety initiatives may be observed. Addressing these gaps requires both systemic investment and cultural change in how women's rugby union is represented and supported through inclusive policy, tailored surveillance systems, and athlete‐centred education (Moore et al. 2024; Stodter and Dane 2024). Recent efforts by governing bodies, including World Rugby, to implement more equitable funding reflect a growing commitment to valuing the women's game and player welfare. This has not yet filtered down into community‐level perceptions of NGBs where the women's game is still seen as a lower priority. To address this inequity, NGBs could improve transparency around funding allocations and actively promote their work in women's rugby, clearly identifying female‐specific initiatives where relevant. Encouragingly, this shift in engagement aligns with World Rugby's growing commitment to advancing female‐focused research and development, as reflected in strategic initiatives like Impact Beyond 2025 and the Blueprint for Growth—Women's Rugby, which aim to elevate visibility, participation, and investment in the women's game globally (Mkumbuzi 2024; World Rugby 2025).

### 4.3 Limitations

This study presents several limitations that warrant consideration. First, its focus on female community‐level rugby union players may constrain the generalisability of findings. However, this sample represents a population that remains critically under‐researched. Within these contexts, injury surveillance practices are typically informal and constrained by limited resources. Resulting from this, the present study utilises an exploratory approach, reflected in the statistical analyses. Findings should be interpreted accordingly. Examining this group provides an important opportunity to generate evidence that can inform and strengthen policy and practice at the grassroots level. Second, the cross‐sectional design captures a single point in time, limiting the ability to assess changes in motivation or engagement with injury surveillance practices longitudinally. While this restricts causal inference, the study provides a foundational understanding of current attitudes and behaviours, which can inform future longitudinal or intervention‐based research.

The reliance on self‐reported survey data introduces potential reporting biases, including recall error and social desirability effects, as well as possible misinterpretation of survey items. These factors may have influenced the accuracy of responses, particularly for behavioural and motivational constructs. In addition, recruitment via social media and professional networks may have introduced sampling bias, potentially over‐representing individuals who are more engaged with the sport or with injury surveillance practices. Nonetheless, self‐report methods remain widely used in behavioural research, particularly for examining perceptions and motivations, and the use of validated instruments helps to partially mitigate these limitations. Finally, we acknowledge that the psychological processes underpinning engagement in player welfare behaviours, such as injury reporting or data collection, may differ from those underpinning motivation to participate in sport.

## 5 Conclusion

This study highlights the complex interplay between motivation to play rugby, experience, and structural support in contributing to engagement with injury surveillance in women's community rugby. Players who expressed more positive attitudes towards injury surveillance and broader sport development were more likely to engage with injury surveillance, while perceived organisational barriers and gender disparities limited participation. Findings underscore the need for athlete‐centred, context‐specific strategies that reflect players' developmental stage and motivational profiles. Addressing systemic barriers, through inclusive policy, tailored education, and female‐specific data systems, is essential to improving player welfare and advancing overall evidence‐based practices, such as injury surveillance, in women's rugby union.

## Funding

M.M.R., E.J., I.S.M. received funding from the Welsh Rugby Union.

## Ethics Statement

This research received ethical approval from Cardiff Metropolitan University (Approval number: Sta‐9801).

## Conflicts of Interest

The authors declare no conflicts of interest.

## Supporting information


Supporting Information S1

## Data Availability

The data that support the findings of this study are available from the corresponding author upon reasonable request.
