# Supplementary material for: Context is Queen: The Good, the Bad and the Rugby: Understanding Engagement With Injury Surveillance in Women's Rugby Union
Source: Eur J Sport Sci. 2026 Sep 16;26(10):e70230. doi: 10.1002/ejsc.70230 (PMC13582287; doi:10.1002/ejsc.70230)
Supplement: Supplementary file 1 [file EJSC-26-e70230-s001.docx]

Supplementary Information

All statistical analyses were performed using SPSS Statistics (Version 29.0; IBM Corp., Armonk, NY, USA), with significance set at *p* < 0.05.

**Regressions**

Linear regression analysis was employed to examine associations between playing experience and motivation-related variables, with all variables entered simultaneously. Prior to analysis, multicollinearity was satisfactory, with all tolerance values > 0.1 and VIF < 10. Inspection of the normal P-P plot indicated that residuals were approximately normally distributed. Only significant predictors are reported. The variables entered were:

- Intrinsic (1 – Low, 2 – High)
- Integrated (1 – Low, 2 – High)
- Identified (1 – Low, 2 – High)
- Introjected (1 – Low, 2 – High)
- External (1 – Low, 2 – High)
- Amotivation (1 – Low, 2 – High)
- Total Motivation Score (1 – Low, 2 – High)

The dependent variable was Playing Experience (continuous).

Binary logistic regression was used to identify predictors of injury surveillance collection**.** Prior to analysis, assumptions were assessed and satisfied: no multicollinearity (tolerance > 0.1, VIF < 10), linearity of the logit confirmed (Box-Tidwell, p = 0.876), and adequate model fit (likelihood ratio p = 0.002, Pearson goodness-of-fit p = 0.998). Twelve influential cases were identified via Cook's distance and are acknowledged as a limitation. The variables entered into the regression model were:

- Playing Experence (continuous)
- Start Playing – Where did you start playing rugby (1 – Club<18, 2 – Club>18, 3 – School, 4 – University)
- Coach Present – Is there a coach present at team training (1 – No, 2 – Yes, 3 – Sometimes)
- Coach – Whos is your is your coach? (1 – Independent, 2 – Players’ partner, 3 – Team member, 4 – Men’s player)
- Coach Qualification – What is the qualification level of your coach? (1 – Unsure, 2 – Level 1, 3 – Level 2, 4 – Level 3, 5 – Level 4-5, 6 – Coaching Degree, 7 – Pro Player, 8 – None)
- Coach Match – Is there a coach present at your team matches? training (1 – No, 2 – Yes, 3 – Sometimes)
- MedAtTraining – Is there medical personnel at your team training? training (1 – No, 2 – Yes, 3 – Sometimes)
- MedAtMatches – Is there medical personnel at your team matches? training (1 – No, 2 – Yes)
- SCCoach – Does your team have a strength and conditioning coach? training (1 – No, 2 – Yes, 3 – Unsure)
- SCPorgramme – Does your team follow a strength and conditioning plan? (1 – No, 2 – Yes)
- SCOwnTime – Do you do any strength and conditioning work in your own time? (1 – No, 2 – Yes)
- MultipleRoles – Do you have multiple roles within your team? (1 – No, 2 – Yes)
- HealthConcerns – Do you think female-specific health concerns are important to your Union/National Governing Body? (1 – No, 2 – Yes)
- FemaleWelfare –– Do you think female-specific player welfare are important to your Union/National Governing Body? (1 – No, 2 – Yes)
- TimeLoss - Do you think time-loss injuries such as concussion, ankle sprains are of concern for your governing body? (1 – No, 2 – Yes)
- NonTimeLoss - Do you think non-time loss injuries such as leaking urine, beast soreness are of concern for your governing body? (1 – No, 2 – Yes)
- Intrinsic (1 – Low, 2 – High)
- Integrated (1 – Low, 2 – High)
- Identified (1 – Low, 2 – High)
- Introjected (1 – Low, 2 – High)
- External (1 – Low, 2 – High)
- Amotivation (1 – Low, 2 – High)
- Total Motivation Score (1 – Low, 2 – High)

The dependent variable was ‘Collect IS’ (Whether they collect Injury Surveillance or not), with the reference category as No/Unsure.
